# Simvastatin Improves Benign Prostatic Hyperplasia: Role of Peroxisome-Proliferator-Activated Receptor-γ and Classic WNT/β-Catenin Pathway

**DOI:** 10.3390/ijms24054911

**Published:** 2023-03-03

**Authors:** Zhen Wang, Shu Yang, Yan Li, Yongying Zhou, Daoquan Liu, Jianmin Liu, Michael E. DiSanto, Xinhua Zhang

**Affiliations:** 1 Department of Urology, Zhongnan Hospital of Wuhan University, 169 Donghu Road, Wuhan 430071, China; 2 Department of Surgery and Biomedical Sciences, Cooper Medical School of Rowan University, Camden, NJ 08103, USA

**Keywords:** benign prostatic hyperplasia, simvastatin, peroxisome-proliferator-activated receptor γ, WNT/β-catenin pathway

## Abstract

Benign prostatic hyperplasia (BPH) is a common disease in elderly men with an uncertain etiology and mechanistic basis. Metabolic syndrome (MetS) is also a very common illness and is closely related to BPH. Simvastatin (SV) is one of the widely used statins for MetS. Peroxisome-proliferator-activated receptor gamma (PPARγ), crosstalking with the WNT/β-catenin pathway, plays important roles in MetS. Our current study aimed to examine SV-PPARγ-WNT/β-catenin signaling in the development of BPH. Human prostate tissues and cell lines plus a BPH rat model were utilized. Immunohistochemical, immunofluorescence, hematoxylin and eosin (H&E) and Masson’s trichrome staining, construction of a tissue microarray (TMA), ELISA, CCK-8 assay, qRT-PCR, flow cytometry, and Western blotting were also performed. PPARγ was expressed in both prostate stroma and epithelial compartments and downregulated in BPH tissues. Furthermore, SV dose-dependently triggered cell apoptosis and cell cycle arrest at the G0/G1 phase and attenuated tissue fibrosis and the epithelial–mesenchymal transition (EMT) process both in vitro and in vivo. SV also upregulated the PPARγ pathway, whose antagonist could reverse SV produced in the aforementioned biological process. Additionally, crosstalk between PPARγ and WNT/β-catenin signaling was demonstrated. Finally, correlation analysis with our TMA containing 104 BPH specimens showed that PPARγ was negatively related with prostate volume (PV) and free prostate-specific antigen (fPSA) and positively correlated with maximum urinary flow rate (Qmax). WNT-1 and β-catenin were positively related with International Prostate Symptom Score (IPSS) and nocturia, respectively. Our novel data demonstrate that SV could modulate cell proliferation, apoptosis, tissue fibrosis, and the EMT process in the prostate through crosstalk between PPARγ and WNT/β-catenin pathways.

## 1. Introduction

Benign prostate hyperplasia (BPH), characterized by unregulated hyperplasia of the periurethral prostate gland, is the most common disease among elderly men, increasing sharply with age, from 50% in men older than 50 years to almost 80% in men aged over 80 years old [1,2]. BPH contributes to lower urinary tract symptoms (LUTSs). Both BPH and LUTSs greatly impact quality of life (QoL) and lead to severe financial burden [3,4,5]. It is known that BPH is closely associated with aging and androgen levels [6]. Although the molecular mechanism of BPH has been extensively studied, including the imbalance between cell proliferation and apoptosis, stromal–epithelial interactions, abnormal sex hormone ratio, cytokines and growth factors, autoimmune and inflammation, stem cells, and oxidative stress (OS) [7,8], the exact pathogenesis remains unclear. In recent years, numerous studies have shown that metabolic syndrome (MetS) is closely related to BPH/LUTSs [9,10,11].

With the improvement of people’s living standards, the incidence of MetS has increased tremendously [12]. A sedentary lifestyle, higher socioeconomic status, high waist circumference, and central obesity are significantly attributed to the development of MetS [13,14], consisting of dyslipidemia, hypertension, hyperglycemia, and visceral obesity [15]. In addition, Hammarsten et al. proposed that BPH could be viewed as a novel aspect of MetS [16]. Indeed, 48.59% of MetS patients are complicated with BPH (MetS-BPH), including its onset, occurrence, and progression [17,18]. Actually, Vignozzi et al. found that rabbits with MetS developed dyslipidemia, increased the accumulation of visceral fat, and decreased gonadotropin, excessive estrogen, and prostatic inflammation [19]. They also proposed that BPH may be a novel metabolic disease [20]. In addition, several studies have revealed that dyslipidemia plays a crucial role in prostate cell proliferation and differentiation and prostate volume change, suggesting that MetS can be used as an independent predictor of BPH/LUTS [21,22,23], especially for total prostate volume > 40 mL [24]. In contrast, high-density lipoprotein cholesterol levels were negatively correlated with total prostate volume, transition zone volume, and intravesical prostatic protrusion [25].

Statins, such as simvastatin (SV), are widely used to suppress cholesterol synthesis by inhibiting 3-hydroxy-3-methylglutaryl-Coenzyme A (HMG-CoA) reductase activity. In addition to lowering cholesterol, statins have anti-proliferative, pro-apoptotic, anti-fibrotic, anti-inflammatory, antioxidant, and protective effects on the vascular endothelium [26,27,28,29]. Indeed, statins have been shown to improve the prognosis of BPH [30]. A prospective study by Zhang et al. [31] showed that SV could significantly reduce prostate volume (PV) and slow the clinical progression of BPH/LUTS. However, the specific mechanism of SV in the development of BPH is still unclear. Moreover, previous studies demonstrated that SV ameliorated heart failure [32] and other cardiovascular events by activating Peroxisome-proliferator-activated receptor gamma (PPARγ)-dependent pathways [33], and modulation of PPARγ signals helps provide pleiotropic protection of SV [34]. PPARγ, a ligand-activated transcription factor and a representative member of the nuclear receptor (NR) superfamily [35], is mainly expressed in adipose tissue and participates in lipid metabolism by selectively enhancing lipid uptake via inducing lipogenesis and increasing lipid storage [36]. More specifically, PPARγ could increase the expression of lipoprotein lipase (LPL), phosphoenolpyruvate carboxykinase (PEPCK), and perilipin, thereby promoting fatty acid storage in adipocytes instead of lipolysis to fatty acid release [37]. PPARγ is activated by binding its ligand-binding domain (LBD), either by a natural ligand (15D-prostaglandin J2, 15D-PGJ2) [38] or a synthetic ligand (thiazolidinedione, TZD) [39]. It then forms heterodimers with the nuclear receptor retinoid X receptor α (RXRα), finally combining with genomic DNA at specific sites [40]. Therefore, it could regulate metabolism, immunity, inflammation, cell differentiation, and cell proliferation [36,41,42]. Several studies have shown that PPARγ plays a crucial role in neural cell differentiation and tumor cell growth [43,44,45]. It was observed that the knockdown of PPARγ in normal human prostate cell lines decreased the expression of prostate differentiation markers [46]. PPARγ can also interfere with prostate cancer cell growth by regulating androgen receptor (AR) levels and activity [47,48,49]. Furthermore, PPARγ is involved in the regulation of the epithelial–mesenchymal transition (EMT) of cancer stem cells and alveolar lipofibroblast [50,51]. Interestingly, PPARγ could reverse pulmonary fibrosis by dedifferentiating myofibroblasts [52].

Finally, it is also known that activation of PPARγ could inhibit classical WNT/β-catenin pathway signaling. On the other hand, WNT/β-catenin pathway activation could lead to PPARγ inactivation in many tissues [53]. Canonical WNT signaling is mediated by the interaction of WNT ligands with specific targets, leading to cytosolic β-catenin aggregation and nuclear translocation, whose nuclear activation triggers the stimulation of downstream factors [54]. Subsequently, it could induce several process, such as cell proliferation, differentiation, apoptosis, inflammation, fibrosis, and EMT [55,56,57,58,59]. Importantly, dysregulation of the classical WNT pathway has been observed in most cancers and other human diseases including BPH [56,58].

In our current study, we investigated the localization of PPARγ in human prostate tissues and the relative expression profiles in normal and hyperplastic prostate tissues. We further treated human prostate cells with SV and a PPARγ inhibitor (GW9662) to detect changes in cell proliferation, cell cycle, apoptosis, fibrosis, and EMT-related markers, and also to detect genes involved in the canonical WNT/β-catenin pathway. In addition, a rat model of BPH established by testosterone supplementation was treated with SV and restored with GW9662 to elucidate the specific role and mechanism of SV in BPH via PPARγ and WNT/β-catenin.

## 2. Results

### 2.1. The Expression and Localization of PPARγ in Human Prostate Tissues and Cell Lines

**Figure 1:**
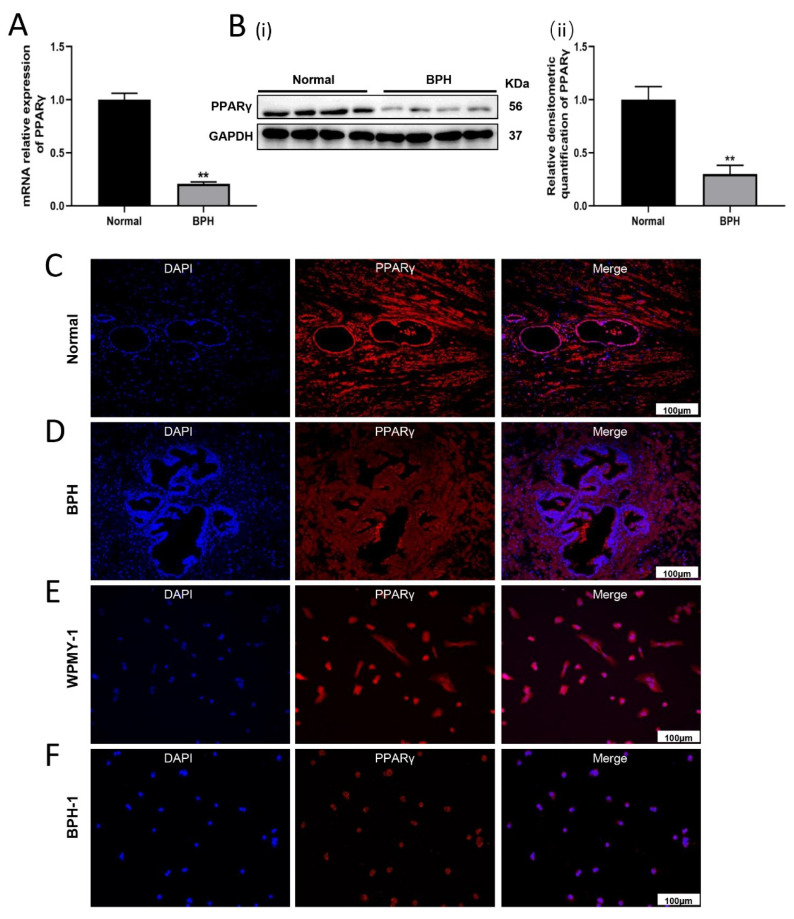
Localization and expression of PPARγ in human prostate tissues and cultured human prostate cell lines. (**A**) Relative expression of PPARγ mRNA levels in human BPH tissues and normal prostate tissues. (**B**) Western blot assay (i) and relative densitometric quantification (ii) of PPARγ proteins in human BPH tissues and normal prostate tissues. (**C**,**D**) Immunofluorescence localization of PPARγ in human normal and hyperplastic prostate. (**E**,**F**) Immunofluorescence staining of PPARγ in human prostate epithelial cells (BPH-1) and stromal cells (WPMY-1). DAPI (blue) indicates nuclear staining and Cy3-immunofluorescence (red) indicates PPARγ protein staining. Sections of all samples were used for immunofluorescence experiments, and representative images were selected. ** *p* < 0.01. Student’s *t* test. The scale bars are 100 μm.

Normal (8 control samples) and hyperplastic prostate tissues (8 BPH samples) were harvested from patients and donors at our institute. The mRNA and protein levels of PPARγ were more than four times and three times higher in the normal prostate than in the hyperplastic prostate, respectively (Figure 1A,B). In addition, immunofluorescence showed that PPARγ was localized in both the epithelial and stromal compartments of the human prostate (Figure 1C,D). Similarly, PPARγ expression was localized in the cytoplasm and nucleus of BPH-1 and WPMY-1 cells.

### 2.2. SV Inhibits Cell Survival by Promoting Cell Apoptosis and Inducing G0/G1 Phase Arrest through PPARγ

**Figure 2:**
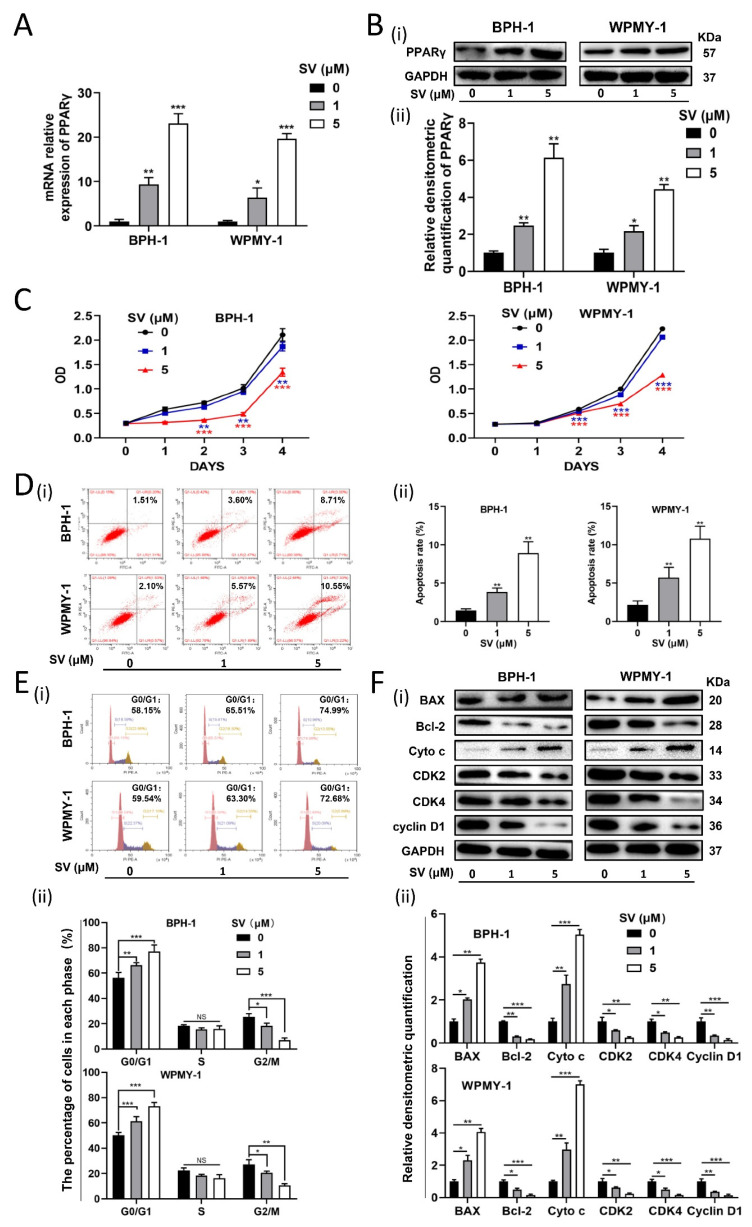
SV inhibited cell survival by promoting cell apoptosis and G0/G1 phase arrest through PPARγ in prostate cells. (**A**) The relative expression of PPARγ at mRNA levels in BPH-1 and WPMY-1 cells treated by simvastatin at 0 μM, 1 μM, and 5 μM for 48 h. (**B**) Western blot assay (i) and relative densitometric quantification (ii) of PPARγ proteins in BPH-1 and WPMY-1 cells treated by simvastatin at 0 μM, 1 μM, and 5 μM for 48 h. (**C**) Cell viability of BPH-1 and WPMY-1 cells treated with different concentrations of simvastatin for 48 h was determined by CCK8 assay. (**D**) Flow cytometric analysis of cell apoptosis in BPH-1 and WPMY-1 cells after simvastatin treated for 48 h. PIPE-A on the y-axis stands for the fluorescence intensity of propidium iodide (PI) and FITC-A on the x-axis stands for the fluorescence intensity of Fluorescein isothiocyanate (FITC) labeled Annexin V (i). The calculation area of the apoptosis rate was percentage of Annexin V+/PI+ cells. Statistical analysis showed the apoptosis rate (%) of BPH-1 and WPMY-1 cells treated with simvastatin (ii). (**E**) Flow cytometry analysis of the cell cycle in BPH-1 and WPMY-1 cells after simvastatin treatment for 48 h (i). Percentages (%) of cell populations at different stages of cell cycles are listed within the panels. Statistical analysis of the percentages (%) of cell populations at different stages of the cell cycles in BPH-1 and WPMY-1 cells after simvastatin treatment (ii). (**F**) Western blot assay (i) and relative densitometric quantification (ii) of apoptosis-related and cell-cycle-related proteins in BPH-1 and WPMY-1 cells after simvastatin treatment. SV = simvastatin, NS means no significant difference, * *p* < 0.05, ** *p* < 0.01, *** *p* < 0.001; one-way ANOVA.

**Figure 3:**
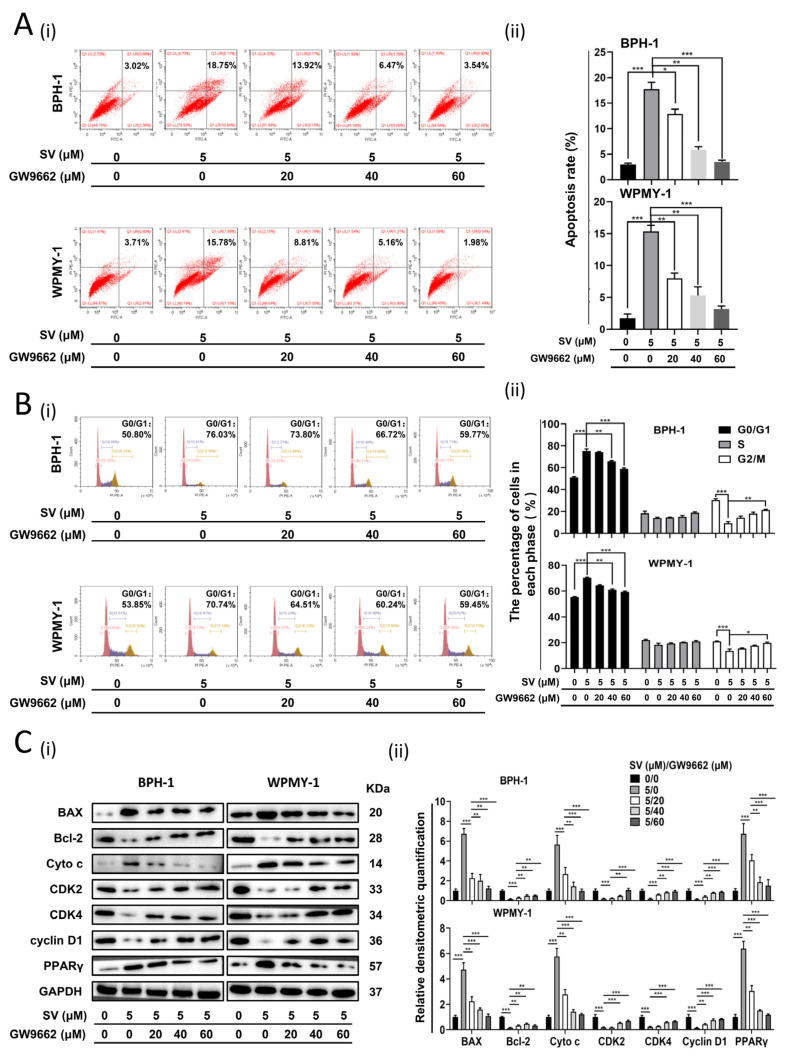
Recovery by GW9662 for cell apoptosis and cell cycle arrest at G0/G1 phase triggered by SV in prostate cells. (**A**) Apoptotic cells staining with Annexin V and PI were revealed by flow cytometry analysis in BPH-1 and WPMY-1 cells treated by GW9662 at 0 μM, 20 μM, 40 μM, and 60 μM for 24 h, and continually treated by simvastatin at 0 μM and 5 μM for 48 h (i). Apoptotic rates (%) were statistically analyzed (ii). (**B**) Flow cytometry analysis of the cell cycle in BPH-1 and WPMY-1 cells after simvastatin and GW9662 treatment (i). Cell cycle arrest at G0/G1 phase by GW9662 was statistically analyzed (ii). (**C**) Western blot assay (i) and relative densitometric quantification (ii) of apoptosis-related and cell-cycle-related proteins in BPH-1 and WPMY-1 cells after simvastatin and GW9662 treatment. Cell types, concentration of simvastatin and GW9662 treatments, and protein masses are indicated. SV simvastatin, * *p* < 0.05, ** *p* < 0.01, *** *p* < 0.001; one-way ANOVA.

Human prostate cells were treated with 1 μM and 5 μM SV. The CCK-8 assay was used to detect cell viability. SV inhibited BPH-1 and WPMY-1 cell survival in a dose-dependent manner with significant differences observed at days 2, 3, and 4 (Figure 2C). In addition, the mRNA and protein levels of PPARγ in both cell lines were dose-dependently elevated (Figure 2A,B). Flow cytometry analysis was used to detect the effects of SV on the cell cycle and apoptosis. As shown in Figure 2D, the percentage of apoptotic BPH-1 and WPMY-1 cells increased significantly at 1 μM (*p* < 0.01) and 5 μM (*p* < 0.01). Furthermore, SV significantly increased the proportion of G0/G1 cells in BPH-1 (*p* < 0.01) and WPMY-1 (*p* < 0.001) cells at 1 μM. More significant differences were shown at higher concentrations of 5 μM (Figure 2E). We further examined related proteins involved in apoptosis and the cell cycle by Western blot, showing a dramatic upregulation of BAX and downregulation of Bcl-2, as well as a significant decrease in proteins involved in the regulation of the G0/G1 phase of the cell cycle (CDK2/4 and Cyclin D1). Moreover, we also found an increase in Cyto c, indicating that SV can promote prostate cell apoptosis in a mitochondria-dependent manner (Figure 2F). Subsequently, both cells were preincubated with/without 20, 40, and 60 μM PPARγ antagonist GW9662 and then treated with 5 μM SV. Consistently, 5 μM SV resulted in cell apoptosis and triggered cell cycle arrest at the G0/G1 phase in BPH-1 and WPMY-1 cells (Figure 3A,B), which were reversed in a dose-dependent manner by pretreating with PPARγ antagonist GW9662 (Figure 3A,B). In addition, both cell-cycle- and apoptosis-related protein alterations induced by SV were significantly reversed by GW9662 (Figure 3C). Additionally, SV-mediated upregulation of PPARγ was reversed by its antagonist GW9662 (Figure 3C).

### 2.3. SV Attenuates Fibrosis and EMT Process in Prostate Cells through PPARγ Pathway

**Figure 4:**
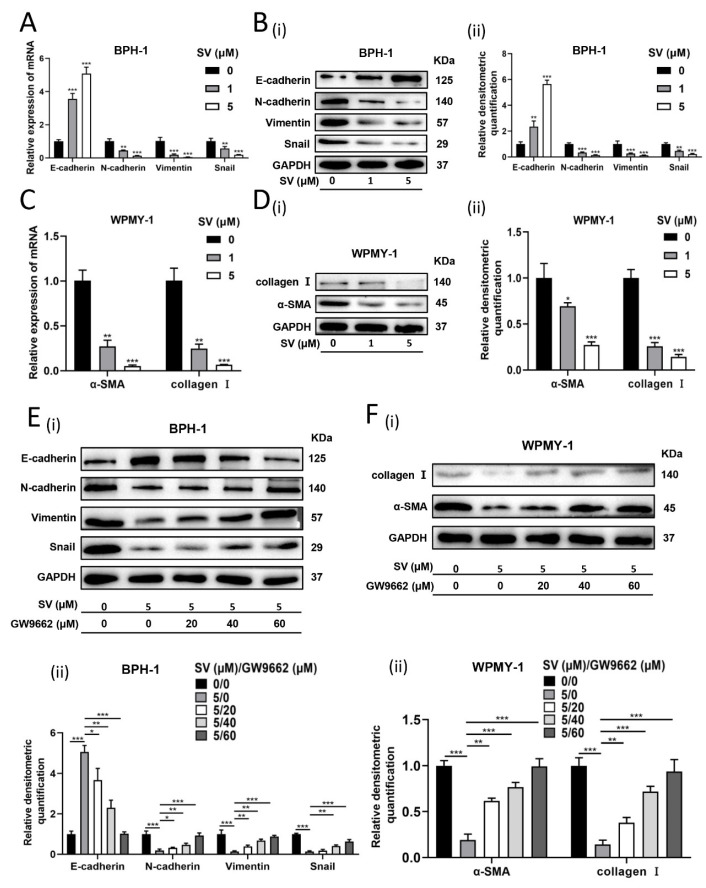
SV inhibited EMT in BPH-1 cells and fibrosis in WPMY-1 cells through PPARγ. (**A**) The relative mRNA levels of EMT markers in BPH-1 cells after simvastatin treatment for 48 h. (**B**) Western blot assay (i) and relative densitometric quantification (ii) of EMT-related proteins in BPH-1 cells after simvastatin treatment for 48 h. (**C**) The relative mRNA level of fibrosis markers α-SMA and collagen I in WPMY-1 cells after simvastatin treatment for 48 h. (**D**) Western blot assay (i) and relative densitometric quantification (ii) of fibrosis markers α-SMA and collagen I in WPMY-1 cells after simvastatin treatment for 48 h. (**E**) Western blot assay (i) and relative densitometric quantification (ii) of EMT-related proteins in BPH-1 cells after simvastatin and GW9662 treatment. (**F**) Western blot assay (i) and relative densitometric quantification (ii) of fibrosis markers α-SMA and collagen I in WPMY-1 cells after simvastatin and GW9662 treatment. SV = simvastatin, * *p* < 0.05, ** *p* < 0.01, *** *p* < 0.001; one-way ANOVA.

We further investigated the effect of SV on relevant indicators of EMT and fibrosis in prostate cells. As shown in Figure 4A,B, in SV-treated BPH-1 cells, it was observed that E-cadherin expression was amplified, whereas N-cadherin, Vimentin, and Snail were significantly lowered at both mRNA and protein levels. In addition, SV significantly suppressed the mRNA and protein expression of fibrosis markers α-smooth muscle actin (α-SMA) and collagen I in WPMY-1 cells (Figure 4C,D). Again, PPARγ antagonist GW9662 significantly rescued the aforementioned changes produced by SV (Figure 4E,F).

### 2.4. WNT/β-Catenin Pathway Crosstalks with PPARγ in Prostate Cells

**Figure 5:**
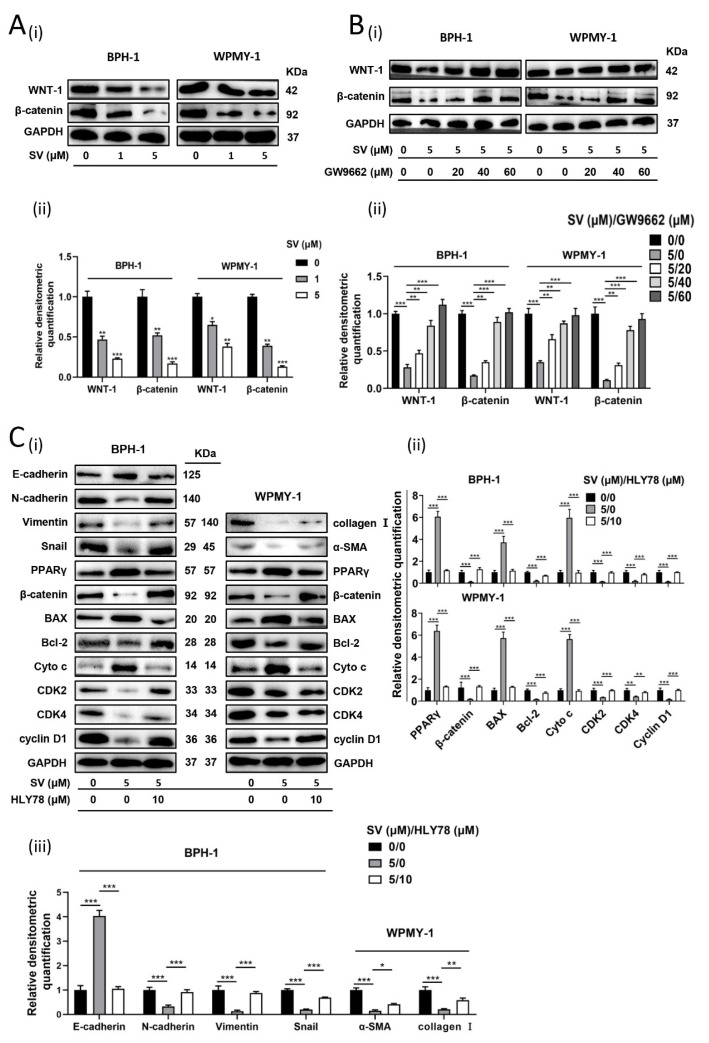
The WNT/β-catenin pathway was inhibited by SV in prostate cells and crosstalks with PPARγ. (**A**) Western blot assay (i) and relative densitometric quantification (ii) of WNT-1 and β-catenin in BPH-1 and WPMY-1 cells after simvastatin treatment for 48 h. (**B**) Western blot assay (i) and relative densitometric quantification (ii) of WNT-1 and β-catenin in BPH-1 and WPMY-1 cells after simvastatin and GW9662 treatment. (**C**) Western blot assay (i) and relative densitometric quantification (ii and iii) of β-catenin, PPARγ apoptosis-related proteins, cell-cycle-related proteins, EMT-related proteins, and fibrosis markers in BPH-1 and WPMY-1 cells treated by WNT/β-catenin pathway-agonist HLY78 at 10 μM for 24 h, and continually treated by simvastatin at 0 μM and 5 μM for 48 h. Cell types, concentration of simvastatin treatment, and protein masses are indicated. SV simvastatin, * *p* < 0.05, ** *p* < 0.01, *** *p* < 0.001; one-way ANOVA.

The WNT/β-catenin pathway is closely related to PPARγ as a fundamental signaling pathway with a wide range of effects. SV dose-dependently attenuated the protein levels of WNT-1 and β-catenin in BPH-1 and WPMY-1 cells (Figure 5A). On the other hand, this inhibitory state generated by 5 μM SV could be abolished by PPARγ antagonist GW9662 in a concentration-dependent manner (Figure 5B). Moreover, 10 μM WNT/β-catenin pathway activator HLY78 could restore the SV-induced alterations of proteins (E-cadherin, BAX, and Cyto c were upregulated; N-cadherin, Vimentin, Snail, α-SMA, collagen I, Bcl-2, CDK2/4, Cyclin D1 were downregulated) (Figure 5C). Interestingly, PPARγ upregulated by SV was similarly rescued with pretreatment of HLY78, maintaining a negative correlation with β-catenin (Figure 5C).

### 2.5. SV Suppressed BPH by Increasing PPARγ In Vivo

**Figure 6:**
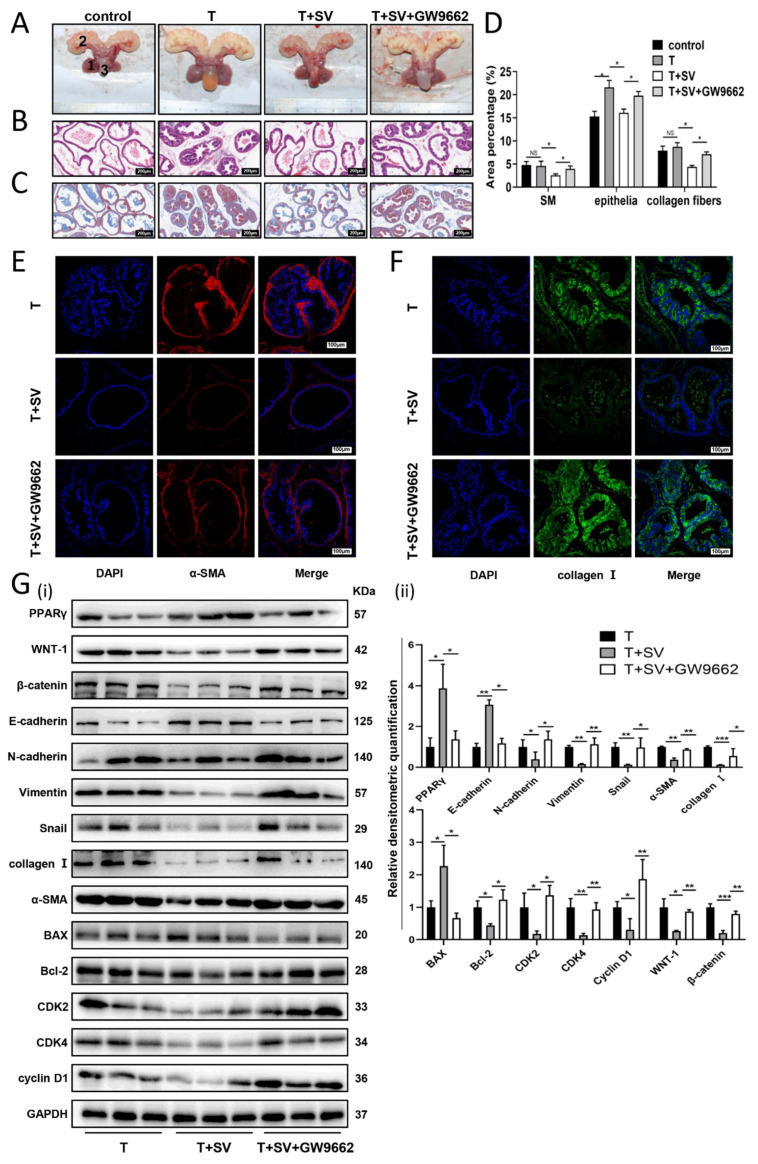
SV inhibited BPH progression and WNT/β-catenin pathway through PPARγ in vivo. (**A**) The rat urogenital tissues from control, BPH, simvastatin-treated, and simvastatin + GW9662-treated rats. (1) Ventral prostate, (2) seminal vesicle, and (3) bladder. (**B**) Representative H&E staining of control, BPH, simvastatin-treated, and simvastatin + GW9662-treated rat prostates. (**C**) Masson’s trichrome staining of control, BPH, simvastatin-treated, and simvastatin + GW9662-treated rat prostates; prostate epithelial cells were stained orange, SM cells were stained red, and collagen fibers were stained blue. (**D**) Percentage of area (%) of different components in Masson’s trichrome staining. (**E**,**F**) Representative immunofluorescence staining of α-SMA and collagen I in prostate tissue from BPH, simvastatin-treated, and simvastatin + GW9662-treated rats. DAPI (blue) showing nuclear staining, Cy3 immunofluorescence (red) showing α-SMA protein staining, and Cy3 immunofluorescence (green) showing collagen I protein staining, and merged images are shown. (**G**) Western blot assay (i) and relative densitometric quantification (ii) of PPARγ, WNT-1, β-catenin, apoptosis-related proteins, cell-cycle-related proteins, EMT-related proteins, and fibrosis markers in BPH, simvastatin-treated, and simvastatin + GW9662-treated rats. *n* = 3 for each group. T = Testosterone propionate, SV = simvastatin, * *p* < 0.05, ** *p* < 0.01, *** *p* < 0.001; one-way ANOVA. The scale bars are 200 μm (panels (**B**,**C**)) and 100 μm (panels (**E**,**F**)).

The BPH rat model was demonstrated by an increase in the weight of androgen-sensitive organs (1.8-fold and 2-fold increase in the ventral prostate and seminal vesicle, respectively) (Figure 6A and Supplementary Table S1, *p* < 0.01). Meanwhile, the body weight of BPH rats was significantly reduced (Supplementary Table S1, *p* < 0.01). The Prostate index (prostate wet weight (mg)/body weight (g)) of BPH rats was 2.5-fold higher than that of controls (Supplementary Table S1, *p* < 0.01). The body weight and ventral prostate weight of the SV-treated rats were decreased by 10.8% and 23%, respectively, and the prostate index decreased by 26.7% (Figure 6A and Supplementary Table S1, *p* < 0.01). When GW9662 was administered 15 min before each SV treatment, it was found that the body weight and ventral prostate weight of the SV-treated rats increased by 1.1-fold and 1.2-fold, respectively, and the prostate index increased by 1.4-fold (Figure 6A and Supplementary Table S1, *p* < 0.05). Triglycerides and cholesterol in rat serum were measured by ELISA. SV can significantly reduce the serum concentrations of triglyceride and cholesterol, and GW9662 can reverse these changes to a certain extent (Figure S1A,B, *p* < 0.001). H&E and Masson trichrome staining were used to observe the histopathological differences of rats in different treatment groups. In BPH rats, BPH mainly occurred in the epithelium, and epithelial cells within the gland are multilayered or with protrusion into the lumen of the papillary lobe. Nevertheless, SV blocked the progression of BPH induced by testosterone. Atrophied glands are lined with a single columnar epithelium to low cubic cells with mild edema. GW9662, in turn, counteracted the effect of SV (Figure 6B). Masson trichromic staining further confirmed that BPH mainly occurred in the epithelium (1.4-fold increase in epithelial cells, *p* < 0.05), while there was no significant difference in stromal components in BPH rats. Interestingly, the stromal component was lower (SM loss of 44.3%, collagen loss of 49.8%) in the SV-treated BPH rats, besides the epithelial component being reduced by 25.5%. In GW9662-plus-SV-treated BPH animals, the epithelial component was elevated by 1.2-fold when compared with the SV-treated group, while the stromal component increased more significantly (SM increased by 1.5-fold, collagen increased by 1.6-fold) (Figure 6C,D). Consistent with our in vitro findings, GW9662 rescued PPARγ that was upregulated by SV in vivo (Figure 6G). The expression of BAX and E-cadherin was upregulated and the expression of α-SMA, collagen I, Bcl-2, CDK2, CDK4, Cyclin D1, N-cadherin, Vimentin, Snail, WNT-1, and β-catenin was downregulated in BPH rats treated with SV. GW9662 significantly ameliorated the above changes induced by SV (Figure 6E–G).

### 2.6. PPARγ/WNT-1/β-Catenin Is Associated with Several Clinical Parameters in Patients with BPH

**Figure 7:**
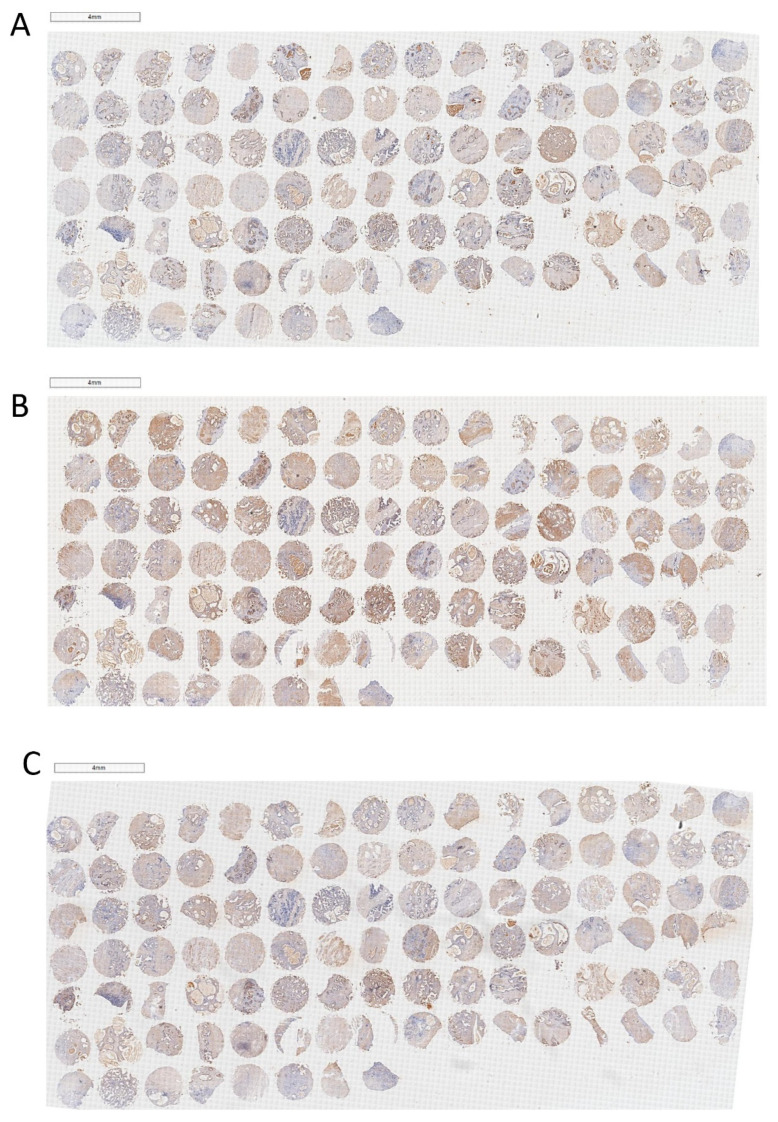
Immunohistochemical analysis of PPARγ, WNT-1, and β-catenin on TMA of BPH. (**A**) PPARγ, (**B**) WNT-1, (**C**) β-catenin. The scale bars are 4 mm.

**Figure 8:**
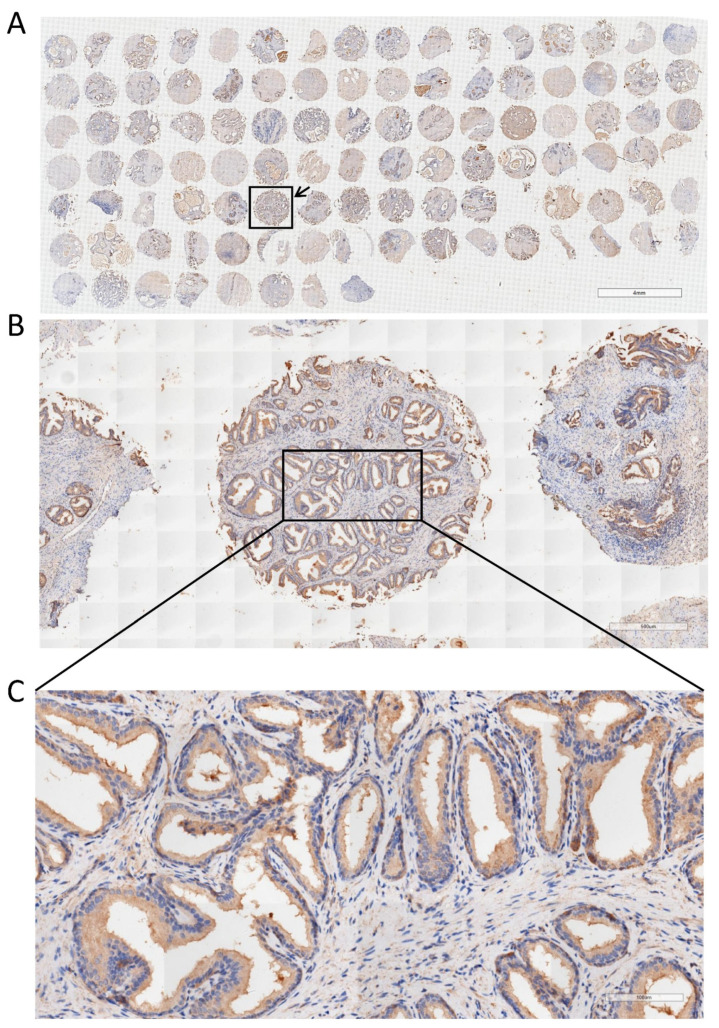
Immunohistochemical analysis of PPARγ on TMA of BPH. (**A**) Immunohistochemical staining of PPARγ in TMA containing 104 BPH samples, the arrow shows a randomly selected tissue sample. The scale bars are 4 mm. (**B**) The enlarged image of the tissue sample selected in A. The scale bars are 500 μm, and enlarge the image in the frame to obtain (**C**) with a scale of 100 μm.

Finally, we obtained BPH tissues from 104 patients and constructed tissue microarrays (TMAs). The percentage of PPARγ, WNT-1, and β-catenin-positive area on the TMA was calculated. As shown in Figure 7 and Figure 8, positive staining for PPARγ, WNT-1, and β-catenin was observed in both the stromal and epithelial compartments of the human prostate. In addition, we analyzed the correlation among the expression of these three proteins in TMA. PPARγ was negatively correlated with β-catenin, but not with WNT-1 (Table 1). As important markers of the WNT/β-catenin pathway, WNT-1 and β-catenin have a very strong positive correlation (Table 1). Meanwhile, we also analyzed the correlation between the expression of these proteins and clinical data. Interestingly, PPARγ was negatively correlated with prostate volume (PV) and free prostate-specific antigen (fPSA) and positively correlated with maximum urinary flow rate (Qmax) (Table 2). WNT-1 and β-catenin exhibited a positive relationship with International Prostate Symptom Score (IPSS) and nocturia, respectively (Table 2).

### 2.7. Overview of SV-PPARγ-WNT/β-Catenin Pathway in BPH

**Figure 9:**
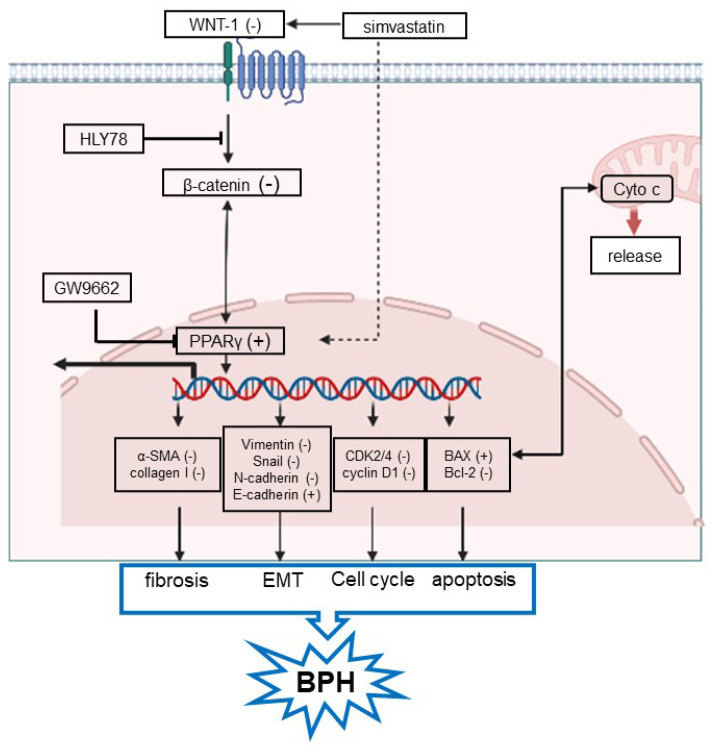
Overview of SV-PPARγ-WNT/β-catenin pathway in benign prostatic hyperplasia. GW9662 = PPARγ antagonist, HLY78 = WNT/β-catenin pathway activator. The red arrow below Cyto c (the right of the image) indicates the release of Cyto c from within the mitochondria to outside the mitochondria. The thick black arrow (left of the image) pointing from DNA outside the nucleus represents post-transcriptional translation of target genes.

As shown in Figure 9, SV can regulate target genes through crosstalk between PPARγ and WNT/β-catenin pathways, such as CDK2, CDK4, cyclin D1, BAX, Bcl-2, N-cadherin, Vimentin, Snail, E-cadherin, α-SMA, and collagen I, which subsequently regulate cell proliferation, cell apoptosis, the cell cycle, EMT, and the fibrosis process.

## 3. Discussion

Our novel data show that PPARγ is localized in the epithelial and stromal compartments of human prostate tissue and is downregulated in hyperplastic prostate tissue. We also demonstrate that SV ameliorates the progression of BPH both in vivo and in vitro through apoptosis, cell cycle arrest at the G0/G1 phase, attenuation of tissue fibrosis, and the EMT process via upregulating PPARγ and inhibiting the WNT/β-catenin pathway.

SV is one of the established statins for the treatment of MetS/hyperlipidemia. Recently, statins were also observed to exhibit therapeutic advantages in patients with BPH/LUTS [31]. Actually, BPH is now regarded as a metabolic disease. As a kind of drug for lowering cholesterol, statins have anti-proliferative, pro-apoptotic, anti-fibrotic, anti-inflammatory, antioxidant, and protective effects on the vascular endothelium [26,27,28,29]. Moreover, it was reported that the PPARγ pathway could contribute to SV, mediating the aforementioned biological process [33]. However, statin-PPARγ signaling had not been completely elucidated, especially for BPH.

PPARγ is a transcription factor belonging to the nuclear receptor superfamily [35]. Genome-wide integration analysis of human-tissue-specific expression [60] has shown that PPARγ is widely expressed in adipose tissue, colon, placenta, bladder, prostate, and other tissues. There are two major isoforms of PPARγ, PPARγ1, and PPARγ2, derived from separate transcription start sites. They are mainly expressed in adipose cells, but PPARγ1 is also expressed at low levels in other tissues, such as the liver, brain, macrophages, and muscle [61,62]. Studies have shown that the expression level of PPARγ1 in human subcutaneous and visceral fat is negatively correlated with obesity, while the expression level of PPARγ2 in human fat is positively correlated with obesity [63]. KEGG (Kyoto Encyclopedia of Genes and Genomes) analysis revealed that the PPARγ1-specific peaks were enriched for pathways that include the PPAR signaling pathway and regulation of lipolysis in adipocytes, while PPARγ2-specific peaks were enriched for the cAMP signaling pathway and vascular smooth muscle contraction pathway [64]. In addition, PPARγ1 plays an important role in cancer. The PPARγ1 ligand can induce G0/G1 phase arrest of human hepatoma cells, and phosphorylation of PPARγ1 can reduce its transcriptional activity and promote the proliferation of human fibrosarcoma cells [65,66]. However, another study has demonstrated that endogenous PPARγ1 could promote ErbB2-mediated breast tumor occurrence and development [67]. Recently, Fang et al. [68] found that PPARγ1 and PPARγ2 regulated the proliferation, apoptosis, and differentiation of proadipocytes in the same way, and the effect of PPARγ2 is more obvious than that of PPARγ1 in the presence of ligands. In conclusion, the specific functions and differences of PPARγ1 and PPARγ2 need to be further studied. Indeed, our current study showed that PPARγ was abundantly expressed in cultured human prostate cell lines, human prostate tissues, and rat ventral prostate. Consistent with a report from Forootan et al. [69], we observed that PPARγ was expressed both in the prostate epithelium and stroma, determined via immunohistochemical staining. Moreover, we found that both the mRNA and protein expression levels of PPARγ were downregulated in the hyperplastic prostate compared with the normal prostate.

To further explore the underlying mechanisms, human prostate cell lines, BPH-1 (epithelial cells) and WPMY-1 (stromal cells), were used and both cell lines were treated with various concentrations of SV. We observed that SV inhibited cell proliferation and caused cell cycle arrest at the G0/G1 phase by reducing the abundance of proteins involved in G0/G1 phase regulation (CDK2/4 and cyclin D1). Simultaneously, upregulation of BAX (a proapoptotic molecule) and downregulation of Bcl-2 (an antiapoptotic molecule) activated intrinsic apoptotic pathways leading to increased apoptosis [70]. This pathway is associated with disruption of the mitochondrial outer membrane potential (MOMP) through BAX activation, leading to the release of cytochrome C from the intermembranous space of mitochondria to the cytoplasm and making cytochrome C further activate BAX [71]. Thus, our data indicated that SV could induce mitochondria-mediated apoptosis. Furthermore, we also observed that SV incubation upregulated the mRNA and protein levels of PPARγ in prostate cells, which was parallel to studies on liver cancer cells and bladder cancer cells [72,73]. In addition to cell growth and death, studies have shown that EMT-involved accumulation of mesenchymal cells was another important process in the development of BPH [74,75,76]. The present study did demonstrate that SV inhibited the EMT process in BPH-1 cells, in which the level of the epithelial marker E-cadherin was upregulated, while the levels of mesenchymal markers N-cadherin, Vimentin, and Snail were downregulated. Moreover, fibrosis could be attributed to BPH progression, which is not responsive to current first-line drugs (α-blockers and 5α-reductase inhibitor) and often leads to surgical intervention. In our current study, we determined that SV incubation decreased the protein expression levels of fibrosis markers α-SMA and collagen I in cultured WPMY-1 cells. Therefore, SV could inhibit cell proliferation, induce apoptosis, and attenuate the EMT and fibrosis process in the prostate.

It has been shown that statins can trigger apoptosis in human lung cancer cells [34], and prevent cell proliferation and EMT in bladder cancer cells through a PPARγ-dependent pathway [73]. To determine whether the aforementioned SV-induced effects on prostate cells were PPARγ-dependent, we pretreated BPH-1 and WPMY-1 cells with the PPARγ-antagonist GW9662. Indeed, SV-induced PPARγ upregulation, increased apoptosis, and attenuated proliferation were significantly reversed by GW9662. This was further supported by the changes in protein levels with increased CDK2/4 and cyclin D1, lowered BAX, and elevated Bcl-2. Moreover, the SV-generated inhibition of EMT and the fibrosis process were also recovered by antagonization of PPARγ. Therefore, the alterations produced by SV could be mediated through PPARγ-dependent signaling.

Additionally, our current study demonstrates that SV-induced upregulation of PPARγ is accompanied by downregulation of the WNT/β-catenin pathway (decreased WNT-1 and β-catenin protein levels). In addition, this change was reversed by pretreatment of cells with GW9662. On the other hand, when cells were pretreated with the WNT/β-catenin agonist HLY78, we observed that SV-induced PPARγ elevation was blocked. Consistently, interactions between PPARγ and WNT/β-catenin pathways had been found in arrhythmogenic right ventricular cardiomyopathy and type 2 diabetes [53]. Moreover, we show that HLY78, like GW9662, can partially reverse the above biological changes generated by SV in prostate cells. Thus, SV could modulate cell growth, cell death, EMT, and fibrosis in prostate cells through crosstalk between PPARγ and the WNT/β-catenin pathway. Similarly, the inverse interaction between the canonical WNT/β-catenin pathway and PPARγ has been demonstrated in many diseases. In cancers, such as glioma and colon cancer, the canonical WNT/β-catenin pathway is upregulated in association with decreased PPARγ expression [77,78]. However, cardiovascular diseases such as cardiac hypertrophy and cardiac overload are diseases in which the canonical WNT/β-catenin pathway is reduced and PPARγ is upregulated [53]. In fact, PPARγ and the WNT/β-catenin pathway interact through a catenin-binding domain within PPARγ and a TCF/LEF β-catenin domain [79,80]. GSK-3β is known to be one of the components of the beta-catenin destruction complex. The phosphatidylinositol 3 kinase/protein kinase B (PI3K/AKT) pathway negatively regulates PPARγ expression through phosphorylation of GSK-3β [81]. In addition, PPARγ agonists activate GSK-3β to reduce β-catenin expression [82]. Therefore, there is a negative correlation between PPARγ and β-catenin, which is consistent with the results of our in vitro and in vivo experiments and TMA. In addition, PPARγ agonists activate Dickkopf-1 (DKK1, a WNT inhibitor) to reduce the canonical WNT/β-catenin pathway [83]. DKK1 can competitively bind lipoprotein-receptor-related protein 5/6 (LPR5/6) to block the associations between WNT-1 and LPR5/6, thus antagonizing WNT signaling [84]. Our in vitro experiments reveal a negative correlation between PPARγ and WNT-1, but TMA shows no significant correlation between them. This may be due to the insufficient sample size of our TMA.

We further translated our in vitro studies into in vivo. Our BPH model induced by testosterone propionate was confirmed by a significant weight increase of the prostate and seminal vesicles when compared with the controls. In addition, SV treatment was validated with significantly lowered serum cholesterol and triglycerides. Similar to our previous observation, a body weight loss was found in BPH rats, which may be due to an increase in daily activities and an increase in lean body mass/fat body mass ratio caused by exogenous testosterone supplementation. Therefore, BPH rats had an even higher relative prostate weight/body weight ratio than that of controls. In addition, consistent with previous studies [85,86], T injection mainly resulted in a significant degeneration of acinar epithelial hyperplasia, such as increased acinar number, protrusion of papillary lobes into the glandular lumen, and thickening of the epithelial layer without a change in the percentage of stromal components. However, the relative prostate weight/body weight ratio of BPH rats was significantly decreased by SV (i.g.) for 28 days. Unlike untreated BPH animals, both epithelial and stromal components of the prostate were reduced in SV-treated BPH rats, which was possibly attributed to SV affecting both the stroma and epithelium. Instead, T mainly showed an influence on the gland. Parallel to our in vitro experiments, GW9662 could significantly reverse the therapeutic effect of SV on the prostate. Moreover, Western blot showed that SV increased the expression of PPARγ, BAX, E-cadherin, and Cyto c and decreased the expression of CDK2, CDK4, cyclin D1, Bcl-2, α-SMA, collagen I, N-cadherin, Vimentin, and Snail. In addition, PPARγ antagonist GW9662 (i.p.) reversed the above changes induced by SV to a certain extent. All these in vivo observations are consistent with our in vitro findings.

Recently, Gong et al. [87] reported that SV alleviated prostatic hyperplasia by alleviating local inflammation in prostate tissue. In their study, the BPH rat model was induced by a high-fat diet and no relevant human tissues and cells data. In addition, their study focused on inflammation-related signaling, instead of PPARγ. Another previous study [88] found that atorvastatin ameliorated T-induced rat BPH. Their study did not provide human data; however, they revealed an inhibitory effect of atorvastatin on oxidative stress markers. Again, their study did not explicitly validate the role of PPARγ. Actually, our study complemented the existing studies by more comprehensively revealing the role and underlying mechanisms of statins in the progression of BPH from human, rat, and cultured cell data.

Finally, the TMA of 104 cases of BPH tissues was constructed, and the correlation among PPARγ, WNT-1, and β-catenin and also their correlation with corresponding clinical data were analyzed. Parallel to our cell and rat studies, it was shown that PPARγ was negatively correlated with β-catenin, but not with WNT-1. In addition, WNT-1 and β-catenin had a very strong positive correlation. Furthermore, we found that PPARγ was negatively correlated with prostate volume (PV) and free prostate-specific antigen (fPSA) but positively correlated with maximum urinary flow rate (Qmax), which is consistent with the inhibitory effect of PPARγ on prostate cell lines. Interestingly, WNT-1 and β-catenin exhibited a positive relationship with International Prostate Symptom Score (IPSS) and nocturia, respectively. Nocturia is reported by a large proportion of untreated patients with BPH and severely affects their quality of life (QoL) [4]. The prevalence of nocturia was positively correlated with age in both men and women. In addition, prostate volume (PV) and prostate-specific antigen (PSA) could be used as predictors of BPH patients who developed bladder outlet obstruction (BOO) [89]. Our findings revealed that PPARγ is negatively associated with the progression of BPH, whereas WNT-1 and β-catenin are involved in promoting the progression of BPH. However, further investigations will be required on the association of PPARγ/Wnt-1/β-catenin with clinical data of BPH patients.

In conclusion, our study demonstrates that PPARγ is expressed in both stromal and epithelial compartments of the prostate and is downregulated in hyperplastic prostate tissues. Our novel data also show that SV could ameliorate the progression of BPH from multiple pathways, including inhibition of cell proliferation, promotion of cell apoptosis and cell cycle arrest at the G0/G1 phase, and inhibition of prostate fibrosis and the EMT process through crosstalk between PPARγ and WNT/β-catenin pathways. Our data suggest that SV may have an ameliorating effect on BPH progression and that the PPARγ-WNT/β-catenin system pathway could play important roles. Further exploration is required to fully elucidate the interaction between the PPARγ and WNT/β-catenin pathway, which may provide new therapeutic targets for the treatment of BPH.

## 4. Materials and Methods

### 4.1. Overview of Common Methods

Cell lines acquisition and culture, CCK-8 assays analysis, flow cytometry analysis, quantitative real-time polymerase chain reaction (qRT-PCR), Western blot analysis, immunofluorescence staining, H&E staining, and Masson trichrome staining were performed as described in our previous studies [90,91,92]. The primer sequences involved are listed in Supplementary Table S2.

### 4.2. Animals and Tissues

Forty-eight specific pathogen-free male Sprague–Dawley rats, aged 6 weeks, weighing 200–250 g, were divided into 4 groups using a random number table method, with 9 rats in each group: Blank control group, Corn oil (MCE, MedChemExpress, China) injection (subcutaneous (s.c.) + intraperitoneal (i.p.)) + dimethyl sulfoxide (DMSO) (MCE, China) administration ((i.p.) + gavage (i.g.)) + PEG300 (MCE, China) administration (i.g.) + normal saline (MCE, China) administration (i.g.); T group, T (Testosterone propionate, Sigma-Aldrich, St. Louis, MO) (2 mg/day)/corn oil injection (s.c.) + corn oil administration (i.p.) + DMSO administration (i.p. + i.g.) + PEG300 administration (i.g.) + normal saline administration (i.g.); T + SV group, T (2 mg/day)/corn oil injection (s.c.) + SV (MCE, China) (20 mg/kg/day)/PEG300/normal saline/DMSO administration (i.g.) + DMSO (i.p.) + corn oil administration (i.p.); T + SV + GW9662 group, T (2 mg/day)/corn oil injection (s.c.) + SV (MCE, China) (20 mg/kg/day)/PEG300/saline/DMSO administration (i.g.) + GW9662 (PPARγ-antagonist, sigma-aldrich, Cat. #M6191) (2 mg/kg/day)/corn oil/DMSO administration (i.p.). SV and GW9662 were dosed according to previous studies [87,93]. Animals were kept at room temperature: 18–22 °C and humidity: 40–60%. On day 28, the rats were euthanized under isoflurane anesthesia (40 mg/kg), and the ventral prostate and seminal vesicles were collected and weighed. Tissues were snap-frozen in liquid nitrogen, followed by storage at −80 °C for subsequent analysis or fixed in 4% paraformaldehyde (PFA) for histological observation. In addition, we collected blood from the rats and left it at 4 °C until the blood clotted to absorb the upper layer of serum, and then stored it at −80 °C for subsequent studies. We were blinded to group assignments when assessing the in vivo effects of SV and GW9662 on the rat prostate. Animal experiments were carried out in the Animal Center of Zhongnan Hospital of Wuhan University and approved by the Medical Ethics Committee for Laboratory Animals of Zhongnan Hospital of Wuhan University.

Prostate tissues were obtained from 8 young brain-dead men who underwent organ donation in Zhongnan Hospital of Wuhan University as controls. The prostate samples of 104 BPH patients with clinical data who underwent transurethral resection of the prostate in the Department of Urology, Zhongnan Hospital of Wuhan University were collected. Postoperative pathological examination confirmed the diagnosis of BPH. Prostate tissues were divided into two parts, which were stored in liquid nitrogen for PCR and Western blot analysis and in 4% PFA for immunofluorescence microscopy and TMA construction. All human specimens were collected and processed in accordance with the guidelines approved by the Ethics Committee of Zhongnan Hospital of Wuhan University and the principles of the Declaration of Helsinki.

### 4.3. The Construction and Immunohistochemical Analysis of TMA

Descriptive statistics of the clinical parameters of 104 BPH patients are presented in Table 3. Tissue from each of the 104 patient cases was fixed, made into donor wax block sections, stained with H&E for pathological diagnosis and lesion tissue localization, and evaluated and confirmed by senior pathologists. A 1.5 mm diameter core was taken from each sample wax block. Finally, we obtained tissue cores from all BPH samples, and the resulting TMA was then serially cut into sections with a thickness of 4 μm. In brief, paraffin sections were dewaxed and placed in citrate buffer (pH 6.0) for antigen repair, followed by blocking of endogenous peroxidase activity in 0.3% H_2_O_2_. Next, the above sections were incubated with the corresponding primary and secondary antibodies (listed in Supplementary Tables S3 and S4, respectively). Antibody localization was identified by addition of peroxidase and 3, 3′-diaminobenzidine tetrahydrochloride. An Olympus DP72 light microscope (Olympus, Japan) was used to image stained sections. Two pathologists blinded to sample type quantified the expression of PPARγ, WNT-1, and β-catenin in prostate tissues derived from the TMA. Image J was used to measure the percentage of positive area for the three proteins.

### 4.4. Drug Treatment of Cells

#### 4.4.1. SV Treatment

BPH-1 and WPMY-1 cells were seeded in six-well or 96-well plates, cultured for 24 h, and treated with SV (0, 1, and 5 μM, dissolved and diluted with DMSO) for 48 h. Subsequent assays were then performed.

#### 4.4.2. GW9662 Treatment

After 24 h of culture, BPH-1 and WPMY-1 cells were pretreated with GW9662 (0, 20, 40 and 60 μM, dissolved and diluted with DMSO) for 24 h, and then treated with SV (5 μM) for 48 h.

### 4.5. Enzyme-Linked Immunosorbent Assay (ELISA)

Triglyceride and cholesterol concentrations in rat serum were measured using Rat Triglyceride, TG ELISA Kit (MEIMIAN, MM-0610R, China) and Rat Cholesterol, CH ELISA Kit (MEIMIAN, MM-0674R, China) according to the manufacturer’s protocol. In brief, 10 μL of rat serum was added to microwell plates embedded with triglyceride or cholesterol antibodies, incubated and washed, and then treated with HRP-labeled antibodies. After another wash, the developer and stop solution were added, and then the absorbance at 450 nm was detected with a microplate reader (Thermo Labsystems, Vantaa, Finland). The triglyceride and cholesterol concentrations in rat serum were then calculated by constructing a standard curve based on the standard concentration.

### 4.6. Statistical Analysis

Data values are expressed as the mean ± standard deviation (SD) of n experiments. In SPSS 20.0, Student’s *t* test was used for comparison between two groups, and one-way ANOVA was used for comparison between multiple groups. *p* < 0.05 was considered significant.

## Figures and Tables

**Table 1:** The correlation analysis among PPARγ, WNT-1, and β-catenin in 104 BPH patients.

	Pearson Correlation Coefficient	*p*-Value
PPARγ vs. WNT-1 PPARγ vs. β-catenin WNT-1 vs. β-catenin	0.1848 −0.2193 0.6525	0.3919 0.0253 * <0.001 ***

* *p* < 0.05 (2-tailed), *** *p* < 0.001 (2-tailed).

**Table 2:** The correlation analysis of clinical parameters and PPARγ/WNT-1/β-catenin in 104 BPH patients.

	PPARγ		WNT-1		β-Catenin	
	Pearson Correlation Coefficient	*p*-Value	Pearson Correlation Coefficient	*p*-Value	Pearson Correlation Coefficient	*p*-Value
Age	0.0177	0.8585	−0.1286	0.1933	0.0307	0.7571
BMI	0.0753	0.4497	−0.0329	0.7417	0.0278	0.7805
PV	−0.2133	0.0297 *	0.0354	0.7212	0.0568	0.5667
tPSA	−0.0578	0.5800	−0.1540	0.1383	0.0386	0.7121
fPSA	−0.3053	0.0025 **	−0.0753	0.4661	0.0024	0.9811
Qmax	0.3125	0.0496 *	−0.0405	0.8042	0.0210	0.8979
RU	0.0738	0.5854	−0.0260	0.8479	0.0417	0.7586
IPSS	0.0649	0.5150	0.2211	0.0248 *	0.1386	0.1626
N	−0.0313	0.7538	0.0740	0.4576	0.2356	0.0166 *

BMI = body mass index, PV = prostate volume, tPSA = total prostate specific antigen, fPSA = free prostate specific antigen, IPSS = international prostate symptom score, Qmax = maximum flow rate, RU = residual urine, N = Nocturia. * *p* < 0.05 (2-tailed), ** *p* < 0.01 (2-tailed).

**Table 3:** Clinical features of 104 patients with benign prostatic hyperplasia.

	Mean	SD
Age (years)	70.13	7.46
Body mass index (kg/m^2^)	22.78	2.76
Prostate volume (cm^3^)	60.82	36.73
Total prostate specific antigen (ng/mL)	7.00	5.89
Free prostate specific antigen (ng/mL)	1.62	1.41
Maximum flow rate (mL/s)	9.97	5.86
Residual urine (mL)	160.66	114.48
International prostate symptom score	21.74	7.37
Nocturia (N)	3.07	1.94

## Data Availability

The data used to support the findings of this study are available from the corresponding author upon reasonable request.

## Supplementary Materials

The following supporting information can be downloaded at: https://www.mdpi.com/article/10.3390/ijms24054911/s1.
