# Supplementary material for: Simvastatin Improves Benign Prostatic Hyperplasia: Role of Peroxisome-Proliferator-Activated Receptor-γ and Classic WNT/β-Catenin Pathway
Source: Int J Mol Sci. 2023 Mar 3;24(5):4911. doi: 10.3390/ijms24054911 (PMC10003121; doi:10.3390/ijms24054911)
Supplement: Supplementary file 1 [file ijms-24-04911-s001.zip › supplementary figure S1.pdf]

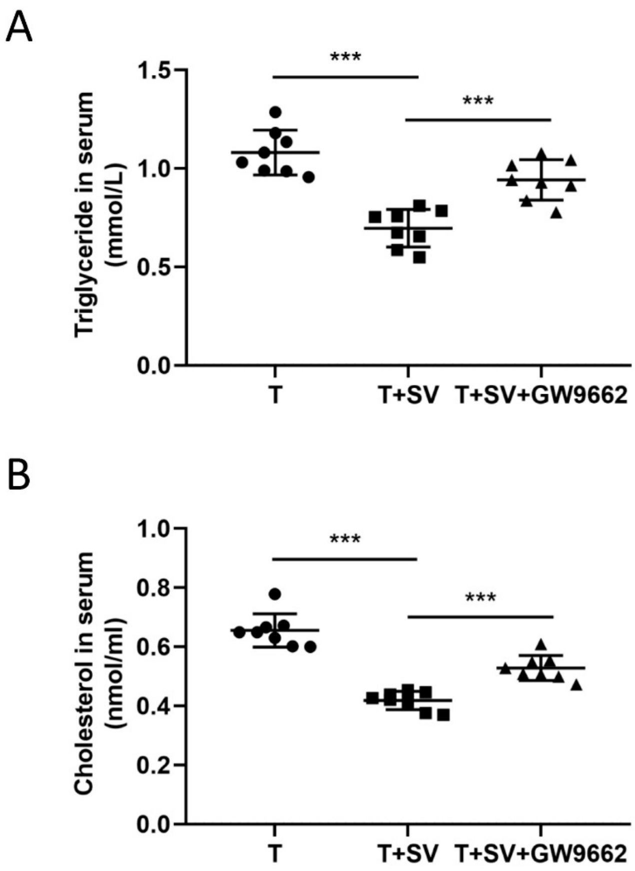

Supplementary Figure legends

Supplementary Fig. S1 Simvastatin reduces lipid concentrations through PPARγ *in vivo*.

(A) Serum triglyceride concentrations were measured by Elisa in BPH, simvastatin-treated, and simvastatin + GW9662-treated rats. (B) Serum cholesterol concentrations were measured by Elisa in BPH, simvastatin-treated, and simvastatin + GW9662-treated rats. T Testosterone propionate, SV simvastatin, *** $p < 0.001$; one-way ANOVA.
