# Supplementary material for: Simvastatin Improves Benign Prostatic Hyperplasia: Role of Peroxisome-Proliferator-Activated Receptor-γ and Classic WNT/β-Catenin Pathway
Source: Int J Mol Sci. 2023 Mar 3;24(5):4911. doi: 10.3390/ijms24054911 (PMC10003121; doi:10.3390/ijms24054911)
Supplement: Supplementary file 1 [file ijms-24-04911-s001.zip › Table S1.pdf]

Supplementary Table S1 Variation of biometric and physiological parameters in Control, T, T+SV and T+SV +GW rats.

Group	Body weight (g)		Ventral prostate Weight (mg)	Seminal vesicles Weight (mg)	Prostate index
	Initial	Final			
Control	219.8(6.3)	505.1(37.5)	683.6(108.2)	1407.4(152.2)	1.2(0.1)
T	223.9(10.0)	410.9(21.6) **	1234.1(129.7) **	2889.6(435.6) **	3.0(0.2) **
T+SV	219.1(9.3)	366.4(14.1) **	950.3(113.3) **	2522.7(387.4)	2.2(0.2) **
T+SV+GW	213.6(5.7)	396.7(10.7) *	1159.6(124.6) *	3202.7(510.3) *	3.1(0.2) *

T testosterone, SV simvastatin, GW GW9662. ** $p < 0.01$ T vs Control, ** $p < 0.01$ T vs T+SV, * $p < 0.05$ T+SV vs T+SV +GW.
