# Supplementary material for: Simvastatin Improves Benign Prostatic Hyperplasia: Role of Peroxisome-Proliferator-Activated Receptor-γ and Classic WNT/β-Catenin Pathway
Source: Int J Mol Sci. 2023 Mar 3;24(5):4911. doi: 10.3390/ijms24054911 (PMC10003121; doi:10.3390/ijms24054911)
Supplement: Supplementary file 1 [file ijms-24-04911-s001.zip › Table S2.pdf]

**Supplementary Table S2** Primer sequence used for qRT-PCR.

| **Target genes** | | **Human（5' to 3'）** |
|---|---|---|
| PPARγ | Forward | AGAGATGCCATTCTGGCCCAC |
| | Reverse | GTGGAGTAGAAATGCTGGAGA |
| E-cadherin | Forward | AATGCCGCCATCGCTTAC |
| | Reverse | TCAGGCACCTGACCCTTGTA |
| N-cadherin | Forward | TCAGGCGTCTGTAGAGGCTT |
| | Reverse | ATGCACATCCTTCGATAAGACTG |
| Vimentin | Forward | GACGCCATCAACACCGAGTT |
| | Reverse | CTTTGTCGTTGGTTAGCTGGT |
| Snail | Forward | GACCACTATGCCGCGCTCTT |
| | Reverse | TCGCTGTAGTTAGGCTTCCGATT |
| α-SMA | Forward | GGCATTCACGAGACCACCTAC |
| | Reverse | CGACATGACGTTGTTGGCATAC |
| collagen I | Forward | GAGGGCCAAGACGAAGACATC |
| | Reverse | CAGATCACGTCATCGCACAAC |
| GAPDH | Forward | ATCCCATCACCATCTTCCAGGAG |
| | Reverse | CCTGCTTCACCACCTTCTTGATG |
