# Supplementary material for: Simvastatin Improves Benign Prostatic Hyperplasia: Role of Peroxisome-Proliferator-Activated Receptor-γ and Classic WNT/β-Catenin Pathway
Source: Int J Mol Sci. 2023 Mar 3;24(5):4911. doi: 10.3390/ijms24054911 (PMC10003121; doi:10.3390/ijms24054911)
Supplement: Supplementary file 1 [file ijms-24-04911-s001.zip › Table S3.pdf]

Supplementary Table S3 List of primary antibodies.

Antigens	Supplier	Species antibodies raised in	Dilution used
PPARγ	ABclonal<br>A0270	Rabbit<br>Polyclonal antibody	1:1000 (WB)<br>1:100 (IF)
BAX	ABclonal<br>A12009	Rabbit<br>Polyclonal antibody	1:1000 (WB)
Bcl-2	ABclonal<br>A11025	Rabbit<br>Polyclonal antibody	1:1000 (WB)
Cyto c	Proteintech<br>10993-1-AP	Rabbit<br>Polyclonal antibody	1:1000 (WB)
CDK2	Cell Signaling<br>Technology, #2546	Rabbit<br>Monoclonal antibody	1:1000 (WB)
CDK4	Cell Signaling<br>Technology, #12790	Rabbit<br>Monoclonal antibody	1:1000 (WB)
Cyclin D1	Cell Signaling<br>Technology, #2978	Rabbit<br>Monoclonal antibody	1:1000 (WB)
E-cadherin	ABclonal<br>A16811	Rabbit<br>Polyclonal antibody	1:1000 (WB)
N-cadherin	ABclonal<br>A0433	Rabbit<br>Monoclonal antibody	1:1000 (WB)
Vimentin	ABclonal<br>A19607	Rabbit<br>Monoclonal antibody	1:1000 (WB)
Snail	ABclonal<br>A5243	Rabbit<br>Polyclonal antibody	1:1000 (WB)
α-SMA	Servicebio<br>GB13044	Mouse<br>Monoclonal antibody	1:1000 (WB)<br>1:500 (IF)
collagen I	ABclonal<br>A1352	Rabbit<br>Polyclonal antibody	1:1000 (WB)<br>1:200 (IF)
WNT-1	Proteintech<br>27935-1-AP	Rabbit<br>Polyclonal antibody	1:1000 (WB)
β-catenin	Proteintech<br>51067-2-AP	Rabbit<br>Polyclonal antibody	1:1000 (WB)
GAPDH	ABclonal<br>AC027	Rabbit<br>Polyclonal antibody	1:10000 (WB)
