# Supplementary material for: Simvastatin Improves Benign Prostatic Hyperplasia: Role of Peroxisome-Proliferator-Activated Receptor-γ and Classic WNT/β-Catenin Pathway
Source: Int J Mol Sci. 2023 Mar 3;24(5):4911. doi: 10.3390/ijms24054911 (PMC10003121; doi:10.3390/ijms24054911)
Supplement: Supplementary file 1 [file ijms-24-04911-s001.zip › Table S4.pdf]

Supplementary Table S4 List of secondary antibodies.

Secondary Detection System Used	Host	Dilution used	Supplier
Anti-Mouse-IgG (H + L)-HRP	Goat	1:10,000 (WB)	Sungene Biotech, Tianjin, China, Cat. #LK2003
Anti-Rabbit-IgG (H + L)-HRP	Goat	1:10,000 (WB)	Sungene Biotech, Cat. #LK2001
Anti-Mouse IgG (H+L), F(ab')2 Fragment (Alexa Fluor® 488 Conjugate)	Goat	1:50 (IF)	Cell Signaling Technology, USA, cat. no. 4408
Anti-Rabbit IgG (H+L), F(ab')2 Fragment (Alexa Fluor® 488 Conjugate)	Goat	1:50 (IF)	Cell Signaling Technology, USA, cat. no. 4412
Hoechst 33342 (1 mg/ml) nucleic acid staining (DAPI)	-	1:750 (IF)	Molecular Probes/Invitrogen, Carlsbad, CA, USA, cat. no. A11007
